# A DNA Repair and Cell Cycle Gene Expression Signature in Pediatric High-Grade Gliomas: Prognostic and Therapeutic Value

**DOI:** 10.3390/cancers13092252

**Published:** 2021-05-07

**Authors:** Natacha Entz-Werlé, Laetitia Poidevin, Petr V. Nazarov, Olivier Poch, Benoit Lhermitte, Marie Pierre Chenard, Hélène Burckel, Eric Guérin, Quentin Fuchs, David Castel, Georges Noel, Laurence Choulier, Monique Dontenwill, Eric Van Dyck

**Affiliations:** 1 UMR CNRS 7021, Laboratory Bioimaging and Pathologies, Tumoral Signaling and Therapeutic Targets, Faculty of Pharmacy, 67401 Illkirch, France; quentin.fuchs2@etu.unistra.fr (Q.F.); laurence.choulier@unistra.fr (L.C.); monique.dontenwill@unistra.fr (M.D.); 2 Pediatric Onco-Hematology Unit, University Hospital of Strasbourg, 67098 Strasbourg, France; 3 ICube-UMR7357, CSTB, Centre de Recherche en Biomédecine de Strasbourg, 67084 Strasbourg, France; l.poidevin@unistra.fr (L.P.); olivier.poch@unistra.fr (O.P.); 4 Multiomics Data Science Research Group, Quantitative Biology Unit, Department of Oncology and Bioinformatics Platform, Luxembourg Institute of Health, L-1445 Luxembourg, Luxembourg; petr.nazarov@lih.lu; 5 Pathology Department, University Hospital of Strasbourg, 67098 Strasbourg, France; benoit.lhermitte@chru-strasbourg.fr (B.L.); Marie-Pierrette.Chenard@chru-strasbourg.fr (M.P.C.); 6 Centre de Ressources Biologiques, University Hospital of Strasbourg, 67098 Strasbourg, France; 7 Paul Strauss Comprehensive Cancer Center, Radiobioly Laboratory, ICANS (Institut de Cancérologie Strasbourg Europe), University of Strasbourg, Unicancer, 67200 Strasbourg, France; h.burckel@icans.eu (H.B.); g.noel@icans.eu (G.N.); 8 Oncobiology Platform, Laboratory of Biochemistry, University Hospital of Strasbourg, 67098 Strasbourg, France; Eric.guerin@chru-strasbourg.fr; 9 Team Genomics & Oncogenesis of Pediatric Brain Tumors, Inserm U981, Gustave Roussy Institute, 94805 Villejuif, France; david.castel@gustaveroussy.fr; 10 DNA Repair and Chemoresistance, Department of Oncology, Luxembourg Institute of Health, L-1526 Luxembourg, Luxembourg

**Keywords:** pediatric high-grade gliomas, DNA damage repair, prognostic clustering, PARP1, XRCC1

## Abstract

**Simple Summary:**

Pediatric high-grade gliomas are incurable brain tumors for which there is a critical need for new therapeutic strategies as well as treatment-predictive biomarkers. This study examined the expression of DNA repair and cell cycle genes in pediatric high-grade gliomas with distinct driving mutations. The aim is to propose a novel classification of these tumors based on sub-groups exposing therapeutic vulnerabilities. Several DNA repair factors were identified that might become new diagnostic markers.

**Abstract:**

Background: Pediatric high-grade gliomas (pHGGs) are the leading cause of mortality in pediatric neuro-oncology, displaying frequent resistance to standard therapies. Profiling DNA repair and cell cycle gene expression has recently been proposed as a strategy to classify adult glioblastomas. To improve our understanding of the DNA damage response pathways that operate in pHGGs and the vulnerabilities that these pathways might expose, we sought to identify and characterize a specific DNA repair and cell-cycle gene expression signature of pHGGs. Methods: Transcriptomic analyses were performed to identify a DNA repair and cell-cycle gene expression signature able to discriminate pHGGs (n = 6) from low-grade gliomas (n = 10). This signature was compared to related signatures already established. We used the pHGG signature to explore already transcriptomic datasets of DIPGs and sus-tentorial pHGGs. Finally, we examined the expression of key proteins of the pHGG signature in 21 pHGG diagnostic samples and nine paired relapses. Functional inhibition of one DNA repair factor was carried out in four patients who derived H3.3 *K27M* mutant cell lines. Results: We identified a 28-gene expression signature of DNA repair and cell cycle that clustered pHGGs cohorts, in particular sus-tentorial locations, in two groups. Differential protein expression levels of PARP1 and XRCC1 were associated to *TP53* mutations and *TOP2A* amplification and linked significantly to the more radioresistant pHGGs displaying the worst outcome. Using patient-derived cell lines, we showed that the PARP-1/XRCC1 expression balance might be correlated with resistance to PARP1 inhibition. Conclusion: We provide evidence that PARP1 overexpression, associated to XRCC1 expression, *TP53* mutations, and *TOP2A* amplification, is a new theranostic and potential therapeutic target.

## 1. Introduction

Despite their low incidence, pediatric high-grade gliomas (pHGGs), including diffuse intrinsic pontine gliomas (DIPGs), are the leading cause of mortality in pediatric neuro-oncology. Resistance to standard therapies is a frequent occurrence in pHGGs [1,2,3]. Recurrent, mutually exclusive mutations affecting K27 (K27M) and G34 (G34R/V) in the N-terminal tail of histones H3.3 and H3.1 act as key biological drivers of pHGGs. These mutations lead to distinct epigenetic reprogramming, telomere maintenance mechanisms, and oncogenesis scenarios, resulting in distinct subgroups of patients characterized by differences in tumor localization, clinical outcome, as well as concurrent epigenetic and genetic alterations [4]. However, contrasting with our understanding of the molecular biology of pHGGs, there has been little improvement in the treatment of pHGGs, for which genotoxic chemotherapy and ionizing radiation (IR) remain mainstays of therapy [1,2,3,5]. In sus-tentorial locations, radiotherapy follows surgical resection, which tends to be extensive, and is associated at least with DNA alkylating agents like temozolomide (TMZ) [6]. In thalamic pHGGs and DIPG, targeted therapies are nowadays proposed concomitantly to irradiation and prolonged until progression [1,2,3,5,7]. Preventing resistance to chemoradiation through the targeting of the underlying pathways is crucial to improve the efficiency of the current treatments. Tumor resistance to irradiation and DNA alkylating agents are promoted in part by complex DNA repair mechanisms orchestrated by the DNA damage response (DDR). These include O^6^-methylguanine-DNA methyltransferase (MGMT) which removes O6-methylguanine (O^6^-meG), the most cytotoxic lesion induced by TMZ, in a direct, suicidal reaction resulting in the transfer of the methyl group to a cysteine residue in the active site of MGMT [8]. Unrepaired O^6^-meG lesions ultimately lead to replication fork collapse and double-stranded DNA breaks (DSBs). As in adult glioblastoma (aGBM) [9], MGMT expression is a crucial biomarker of response to TMZ in pHGG patients [10]. Other DNA repair mechanisms include direct repair mechanisms mediated by the oxidative demethylases ALKBH2/3, as well as the base excision repair (BER) pathway, the mismatch repair (MMR) pathway, and pathways that repair DSBs by homologous recombination (HR) or non-homologous end-joining (NHEJ). These pathways are paramount to preserve genetic integrity and cell survival [11,12,13]. Thus, a hypermutator phenotype resulting from mutations in DNA polymerase genes and defective MMR was found to act as a driving force in a subset of pHGGs [14,15]. MMR defects were also observed following treatment of adult and pediatric HGGs displaying specific gene promoter methylation patterns [16]. Recent studies have associated alterations in DNA damage (DD) repair control with histones and/or p53 mutations. Histone post-translational modifications (PMTs) constitute an important epigenetic determinant of the structure and function of chromatin. Such histone PTMs appear to modulate the DDR [17]. In H3.3 *K27M* DIPG, radioresistance was found to be driven by *TP53* mutations and could be prevented by RNAi-mediated depletion of the serine/threonine-protein kinase CHK1 required for checkpoint-mediated cell cycle arrest and activation of DNA repair in response to DD or unreplicated DNA [18]. In those DIPG, histone H3 demethylase inhibition using GSK-14 enhanced the efficiency of IR by inhibiting DSB repair by HR [19]. In contrast, H3.3 *G34* mutants were found to display increased genomic instability and replicative stress, as well as a defective HR-mediated repair [20,21]. In aGBM, a novel classification was recently achieved using unique DNA repair and cell cycle gene expression signatures exposing vulnerabilities to DNA damaging agents and inhibitors of the DNA damage response [22]. The evidence therefore suggests that targeting DD repair components might contribute to the prevention of therapeutic resistance while offering new therapeutic approaches against pHGGs.

Here, to improve our understanding of the DDR pathways that operate in pHGGs and the vulnerabilities that these pathways might expose, we first challenged gene expression datasets from pHGG cohorts, including DIPG and sus-tentorial tumors, with the DNA repair and cell cycle gene expression signatures initially identified in aGBMs [23]. Through comparison with low-grade pilocytic astrocytomas (LGG), we next identified a novel and specific DNA repair and cell cycle gene expression signature of 28 genes (hereafter referred to as the pHGG signature) that segregated pHGGs from LGGs. Notably, this signature shared key components of the DDR with the aGBM signature. Analysis of selected genes of the pHGG signature at the gene and protein expression levels revealed its correlation to *TP53* mutation and highlighted high PARP1 and XRCC1 protein expressions as immunohistochemical theranostic biomarkers of worst therapeutic response in pHGGs. Finally, we used primary patient-derived cell lines to correlate the PARP-1/XRCC1 expression balance with resistance to PARP1 inhibition.

## 2. Materials and Methods

### 2.1. Pediatric High-Grade Gliomas’ Collection

Twenty-one pHGGs (3 DIPGs, 8 supra-tentorial, and 10 thalamic locations) were collected and included in the study after obtaining written informed consents from parents and patients. Aged from 7 to 19 years, pHGGs were followed from January 2008 to January 2016 at the University Hospital of Strasbourg (UHS). The research protocol was validated by the local institutional ethics committee. They were all screened by Next-Generation Sequencing (NGS) for driver mutations including H3.3 and H3.1 *K27M*, H3.3 *G34V/R*, *IDH1R132H*, and *BRAFv600* mutations as already described [19]. We also explored *TP53* mutations, as well as *PARP1* and *TOP2A* amplifications. Fresh-frozen and paraffin-embedded specimens were obtained from the Centre de Ressources Biologiques of UHS (declaration number DC-2017-3090). MMR deficient patients and gliomatosis cerebri were not included in this cohort. We used formalin-fixed, paraffin-embedded (FFPE) specimens for the tissue-microarray (TMA) construction comprising the 21 diagnostic samples and 9 paired relapses. Six out of the 21 fresh frozen diagnostic tissues were provided for transcriptomic analyses. Ten pilocytic astrocytomas (PAs) treated in our Strasbourg’s center were also selected for omics comparison. All pHGG patients were treated in first line therapy with IR, which was systematically associated to temozolomide except for DIPGs.

For the statistical analyses, we defined 3 classes in our 21-pHGG population, based on the clinical evolution of patients: (1) group 1 comprised patients with a very early relapse, where the progression/relapse was observed within 5 months of diagnosis in DIPG and within 9 months in thalamic and sus-tentorial locations, (2) group 2 comprised patients where the progression/relapse occurred after 6 or 9 months depending on the same respective locations, and (3) group 3 comprised patients with a long-term survival above 36 months.

### 2.2. Patient-Derived Cell Lines (PDCL) Generation

As already described in our previous publication [23], we generated different cell lines from patient biopsies at diagnosis. All lines (BT35, BT69, BT68, and BT83) harbor the H3.3 *K27M* mutation and were derived from 2 thalamic pHGGs and 2 DIPGs, respectively. BT68 and BT69 are cultures as neurospheres and BT35 and BT83 as a monolayer cell line. We also used a commercialized cell line, which is UW479.

### 2.3. Transcriptomics and Biostatistical Analyses

Total RNA was prepared from the 6 pHGG diagnostic samples, 10 Pas, and 4 PDCLs using Trizol reagent (ThermoFisher Scientific, Waltham, MA, USA). RNA concentrations were quantified on a NanoDrop™ 3300 fluorospectrometer (ThermoFischer scientific, Waltham, MA, USA). Gene expression analysis was performed using Affymetrix U133 Plus 2.0 chips. Data were read with the *affy* package and normalized with RMA. Gene expression was annotated with Ensembl genes from build 37 assembly 71. We also used already published DIPG and sus-tentorial pHGGs’ RNA datasets [24,25].

Gene expression analyses were carried out in multiple steps. First, we performed hierarchical clustering on gene-scaled data using *pheatmap* R package with Euclidian distance as a similarity measure. To predict the G1/G3 proliferation status of the pHGG samples, we first combined the aGBM dataset from the Köln cohort [22] (hereby called reference aGBM dataset) with each pHGG dataset, using *ComBat* from *sva* package [26] for batch correction. We then trained a random forest classifier on the G1 and G3 groups of the Köln dataset and verified that it reproduced the tumor classification of our aGBM dataset previously obtained [22]. Finally, we predicted groups for the combined dataset and visualized probabilities generated by a random forest. We compared the performance of our signatures using well-established gene signatures of cell cycle and DNA repair from MsigDB (Cell cycle GSEA (170 genes, https://www.gsea-msigdb.org/gsea/msigdb/cards/CELL_CYCLE_PHASE (accessed on 6 April 2021), DNA repair GSEA (150 genes, https://www.gsea-msigdb.org/gsea/msigdb/cards/HALLMARK_DNA_REPAIR (accessed on 6 April 2021), Reactome_DNA_repair GSEA (332 genes, https://www.gsea-msigdb.org/gsea/msigdb/cards/REACTOME_DNA_REPAIR (accessed on 6 April 2021), as well as the biological process terms “DNA repair” and “cell cycle” from Gene Ontology (GO). We also considered the union of cell cycle and DNA repair signatures as well as DDR-specific subsets included in cell cycle signatures. As an additional approach, we used unsupervised consensus independent component analysis (ICA). Here, the components are defined as statistically-independent signals in the space of genes, whose biological relevance can be assessed using standard functional annotation (e.g., enrichment analysis) [27]. ICA presents the advantage that it simultaneously corrects for confounding factors such as platform effect and disentangles cell type heterogeneity and intra/inter-tumor heterogeneity, thus resulting in clear biological signals. ICA requires larger datasets to be properly applied, therefore we combined presented data with publically available data from TCGA (GBM and LGG cohorts).

To identify a DNA repair and cell cycle gene expression signature of pHGG, a differential analysis was done between the 6 pHGGs of our patient collection and 10 pilocytic astrocytomas. First, principal component analysis (PCA) was performed to assess the consistency of the data. Then, a relative intensity analysis (pHGGs/pilocytic astrocytomas) was performed to obtain fold change (FC). Only genes with higher than 1.3 or lower than −1.3 were considered to have a significant difference. Finally, a gene ontology enrichment analysis was performed using Panther (PANTHER—Gene List Analysis (pantherdb.org)) to detect the underlying biological processes. The pHGG signature was challenged with the various pHGG and PDCL datasets using the analytic approaches described for the aGBM signatures.

### 2.4. TOP2A and PARP1 Gene Expression

Total RNA was prepared from the 21 pHGG specimens studied by IHC using the RNeasy Plus Universal Tissue Mini kit (Qiagen, Hilden, Germany). The mRNA expression levels of *TOP2A* and *PARP1* was then measured by quantitative RT-PCR using the SYBR Green Master I technology on a LightCycler (Roche, France). The results were expressed as 2^−ΔCt sample^/2^−ΔCt control^ = 2^−(ΔCt sample−ΔCt control)^= 2^−ΔΔCt^. *PBGD* (Cy5-labelled forward primer 5′-CTGGTAACGGCAATGCGGCT-3′ and reverse primer 5′-GCAGATGGCTCCGATGGTGA-3′ (amplified product of 328 bp on cDNA)) was used as reference gene [28] and a calibrating pool of normal brain tissue RNA (10 hippocampal scleroses) was used to assess the normal ranges of each gene expression. The primers were targeting mRNA of PARP-1 [29] and *TOP2A* (Cy5-labelled forward primer 5′-GCCATTGGCTGTGGTATTG-3′ and *TOP2A* reverse primer 5′-GAGAAGCTTCTCGAACATTGAG-3′).

### 2.5. TMA Constitution and Immunohistochemistry (IHC)

FFPE tumor samples from patients were gathered on a TMA block using a tissue arrayer (MTA BOOSTER 01 V2.04, Excilone, Elancourt, France) with 3 representative areas selected per tumor. IHC was performed using an automated tissue staining system (Ventana Medical Systems, Inc., Tucson, AZ, USA). The APE1, MPG, XRCC11, RAD51, PARP1, and p53 antibodies were purchased from Abcam (Paris, France) and Ki67 from Dako (clone MIB-1, Dako, Santa Clara, CA, USA). Two pathologists analyzed protein expression independently.

### 2.6. Cell line Characterization, Drug Testing, and Irradiation

PDCL BT35, BT68, BT83, and BT69 were plated in 96-well flat bottom plates (5000 cells/well) in normoxic conditions at 21% of oxygen. Cell growth (% of occupied area) was imaged with an IncuCyte™ ZOOM cell imaging system (Essen BioScience, Ann Arbor, MI, USA) for 10 days and analyzed using the Incucyte Zoom software. Olaparib was used over a concentration range of 0.1 to 100 µM. All drug testing experiments were done in duplicate.

### 2.7. IHC Statistical Analyses and Correlations with Outcome

The GraphPad Prism 7 software was used for box plots generation showing the mean +/− s.e.m. Comparisons between 2 groups were performed using the non-parametric Mann–Whitney test. Comparisons between the 3 groups of therapeutic response were done using the non-parametric Kruskal–Wallis test. Drug inhibition curves and a 50% inhibitory concentration (IC50) calculation were generated with the GraphPad software for all cell lines at 96 h.

SPSS 11.5 was used for Kaplan Meier survival analyses. A *p*-value of <0.05 was considered statistically significant and n.s. was reported for non-statistically significant results (*p* > 0.05). We also did a multivariate comparison of prognostic biomarkers using a Cox regression models.

A heatmap was generated using the Excel software with all tumor characteristics and protein expressions.

## 3. Results

### 3.1. Stratification of Pediatric High-Grade Glioma (pHGG) Using Adult Glioblastoma (aGBM) DNA Damage (DD) Repair Signature

We previously described unique DNA repair and cell cycle gene expression signatures that resulted in the classification of aGBM specimens into two major groups (G1 and G3) displaying inverse expression profiles, and a third less-defined group (G2). Specifically, we originally identified a 52-gene signature that could be refined into a 27-gene signature able to reproduce the initial classification (Figure S1). Of note, our 27-gene signature contained important effectors and regulators of the cell cycle, chromosome segregation, and mitosis (e.g., AURKA, AURKB, CCNA2, CCNB1, CDC25C, CDC6, CDK1, CENPA, CENPF, MKI67, KIAA0101/PCLAF, PLK1, PTTG1, TOP2A), all up-regulated in G3 specimens compared to G1 specimens. It also contained genes encoding crucial HR factors such as the RAD51 recombinase, the chromatin remodelers RAD54B and RAD54L, and enzymes involved in Holliday junction resolution (EME1/MUS81 complex) and/or NER (ERCC3 (XPB), ERCC4 (XPF)). Also in the signature were genes encoding the DNA glycosylase NEIL3, Fanconi Anemia factors (FANCD2, UBE2T), the ubiquitin protein ligase UBE3B, and two specialized DNA polymerases, POLM and POLQ, involved in the non-homologous end-joining (NHEJ) pathways of DSB repair. Consistent with the upregulation of the cell cycle gene component of the 27-gene signature, G3 tumors were highly proliferative compared to G1 tumors. Furthermore, Gene Ontology (GO) analysis of the biological processes that distinguished the G1 and G3 groups indicated that G3 group tumors developed cellular programs to sustain high proliferation (e.g., cell cycle and cell division, chromosome organization and segregation, DNA replication and repair, chromatin assembly). In contrast, G1 tumors engaged critical survival processes (e.g., apoptosis and autophagy, cellular adhesion, response to stress).

**Figure 1:**
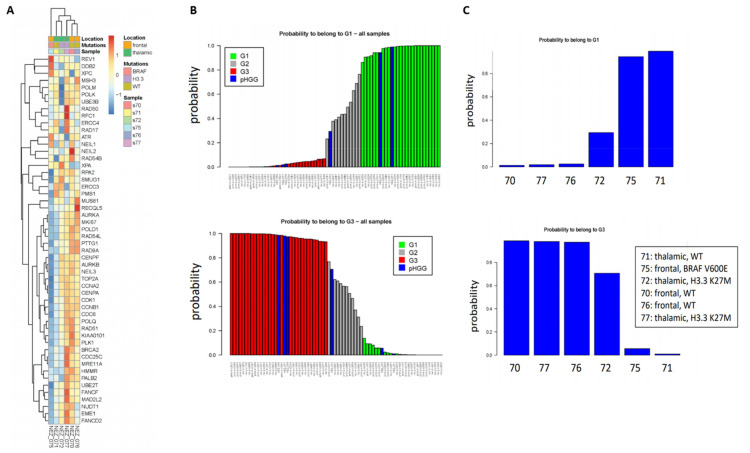
Clustering of pHGG (pediatric high-grade gliomas) using aGBM (adult glioblastoma) DNA repair and cell cycle gene expression signatures. (**A**) Segregation of the indicated pHGG specimens obtained with the 52 gene signature of aGBMs. (**B**,**C**) Prediction of the proliferation status of the pHGG specimens following random forest analysis using the “Köln” cohort of aGBMs of Gobin et al. [22] as training dataset. Green and red bars indicate G1 (less proliferative) and G3 (more proliferative) aGBM samples, respectively. These groups display opposite profiles of the gene expression signature. Grey bars indicate undefined tumors, referred to as G2 in Gobin et al. Following combination with the training dataset (**B**), the probability of each pHGG specimens (indicated by blue bars) to belong to the G1 or G3 group was assessed (**C**).

As a first step to investigate whether our signatures could shed new light in the classification of pHGGs, we studied a minimal set of six pHGG patients composed of wild-type (*wt*) or H3.3 *K27M* thalamic tumors, as well as *wt* or *BRAFv600e* mutated frontal tumors. To this end, and with the goal of exploiting as much as information as possible, we used the 52-gene signature. Indeed, this signature is composed of two components of similar size displaying co- or anti-expression regulation, respectively, in the G1 and G3 groups. As shown in Figure 1A, we observed clusters of samples displaying different profiles of our 52-gene signature. We next predicted the G1/G2/G3 status of the pHGG specimens following combination of the pHGG dataset with our reference aGBM dataset and Random Forest analysis. Our signature predicted robustly pHGG specimens with G1 or G3 status as well as less-defined tumors (G2) (Figure 1B,C). However, no correlation could be established between this status and the H3.3/BRAF mutational status as G1 tumors comprised wt thalamic (n = 1) or frontal mutated BRAF (n = 1), while G3 tumors were *wt* frontal (n = 2) or thalamic mutated H3.3 (n = 1) (Figure 1C). With the caveat that this is a small dataset, these observations suggest that the 52-gene expression signature established in aGBMs can indeed capture features related to cell cycle and DNA repair in pHGG tumors. However, the observed segregation does not correlate with underlying driver mutations affecting histones or BRAF, neither with treatment response groups as defined in Section 2.1.

**Figure 2:**
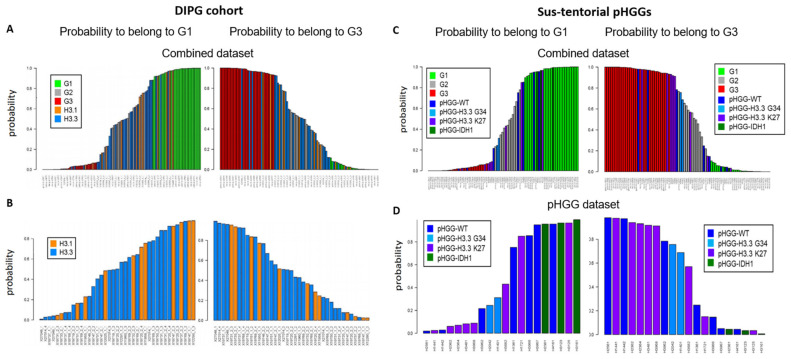
Group prediction in larger cohorts of pHGG using adult DD repair and cell cycle signature. Prediction of the G1, G2, and G3 status of the indicated specimens from a cohort of DIPG (**A**,**B**) or sus-tentorial pHGGs (**C**,**D**). Predictions were obtained by random forest analysis using the Köln cohort of aGBMs as training dataset, as in Figure 1.

We next considered a cohort of DIPG specimens with either H3.1 *K27M* or H3.3 *K27M* mutations [24]. As seen with the previous small pHGG cohort, Random Forest analysis identified G1, G2, and G3 specimens (Figure 2A). However, H3.1 and H3.3 mutant tumors could not be segregated into two separate groups based on our DNA repair and cell cycle gene expression signature (Figure 2B, see also Figure S2A for a heatmap based on the aGBM signature). This observation suggests that the H3.1/H3.3 mutant status is not a determinant of this classification and that the impact of the *K27M* mutation on cell cycle and DNA repair might be similar irrespective of the nature of the histone that carries it (H3.1 or H3.3). To ascertain this notion, we verified that well-characterized signatures of cell cycle and/or DNA repair (see Section 2.3 of Materials and Methods for a description) also failed to distinguish H3.1 from H3.3 mutant tumors (data not shown). We then used ICA. We first combined the H3.1/H3.3 dataset with a TCGA dataset comprising adult GBM (171 samples) and low-grade gliomas (LGGs, 530 samples) to allow a sample size affording robust analysis. We then examined the ability of the “cell cycle component” of ICA to segregate H3.1 and H3.3 tumors into separate groups. However, confirming our previous approaches, no segregation of mutant H3.1 and H3.3 specimens was observed (Figure S2B,C).

We next characterized a cohort of 20 specimens comprising sus-tentorial or thalamic tumors [25]. This pHGG group includes wt pHGGs (n = 7), as well as tumors harboring H3.3 *G34R* (n = 2), H3.3 *K27M* (n = 8), or mutant IDH1 (n = 3). Again, we were able to assign a G1 or G3 status to most tumors while also identifying less-defined tumors (Figure 2C). Notably, all three *IDH1* mutant specimens were G1, whereas a majority of H3.3 *K27M* tumors were G3 (4/7) and the two H3.3 *G34R* tumors had an ill-defined G2 status (Figure 2D). However, wt tumors and to a certain extent H3.3 *K27M* tumors were distributed among the three groups. A heatmap illustrating the segregation of the sus-tentorial specimens based on the aGBM DNA repair and cell cycle gene expression signature is shown in Figure S3.

### 3.2. Stratification of Thalamic and Hemispheric pHGGs with a Pediatric Specific 28-Gene Expression Signature of DNA Repair and Cell Cycle

**Figure 3:**
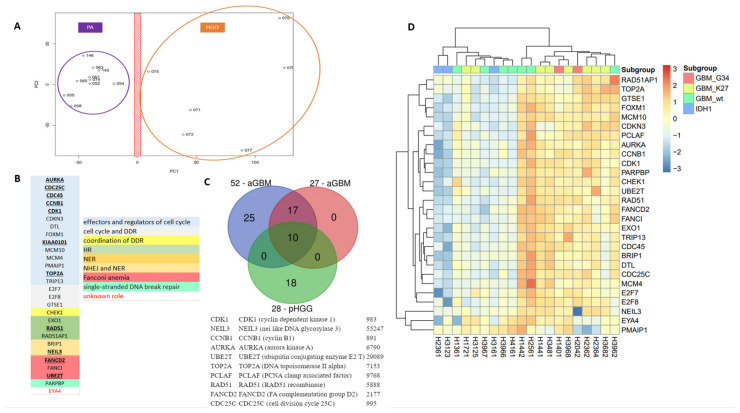
A specific 28-gene DNA repair and cell cycle gene expression signature distinguishes pHGGs from pediatric low-grade gliomas. (**A**) Principal component analysis score plot for pilocytic astrocytoma (PA) samples and pHGG specimens. (**B**) Identity and features of the 28 DNA repair and cell cycle genes composing the signature of pHGGs. Underlined genes are also present in the aGBM signatures of Gobin et al. (**C**) Venn diagram showing how the 28-gene pHGG signature overlaps with the 52- and 27-gene aGBM signatures, and list of the 10 genes shared by these signatures. (**D**) Heatmap illustrating the segregation of sus-tentorial pHGG specimens into 2 groups based on the pediatric DDR gene signature.

As the DNA repair and cell cycle gene expression signatures established in aGBM did not afford segregation of mutant H3.1 and H3.3 specimens, we next sought to determine whether a specific pHGG signature could be identified that afforded such segregation. As a first step to identify a signature of pHGGs, we compared transcriptomic datasets from pHGGs to that of a cohort of low-grade gliomas composed of pilocytic astrocytomas (PAs). Using PCA, we were able to segregate the two populations of tumors based on principal component 1 (PC1) (Figure 3A). Of note, the first two PCs accounted for approximately 45% of the total variance. PAs formed a more compact group than pHGGs, suggesting that the pHGG group was more heterogeneous, in line with the driver heterogeneity in such tumors and the phenotypic diversity. We next identified a list of 185 genes that displayed a 1.3-fold over-expression in pHGGs compared to PAs. GO enrichment analysis of this list revealed major statistical enrichments in GO modules corresponding to cell cycle and DDR processes. We noted the presence in this list of a set of 28 DNA repair and cell cycle genes, hereafter referred to as 28-DD repair genes (Figure 3B). Among this signature were genes involved in cell cycle (AURKA, CCNB1, CDC25C, CDC45, CDK1, CDKN3A, DTL, FOXM1, KIAA0101/PCLAF, MCM10, MCM4, PMAIP1, TOP2A, TRIP13), as well as genes bridging cell proliferation to DD response promotion (CHEK1, E2F7, E2F8, GSTE1), and key components of various DNA repair pathways (EXO1, RAD51, RAD51AP1, PARPBP, FANCI, FANCD2, UBE2T, BRIP1, NEIL3). Notably, this signature shared 10 genes with the aGBM signatures (AURKA, CCNB1, CDC24C, CDK1, KIAA0101/PCLAF, TOP 2A, RAD51, NEIL3, FANCD2, and UBE2T) (Figure 3C).

To validate the novel signature and evaluate its prognostic and theranostic value, we then carried out hierarchical clustering in cohorts of sus-tentorial/thalamic or DIPG pHGGs described above. Importantly, as shown in the heatmap of Figure 3D, the 28-gene DD repair signature clustered clearly the thalamic and hemispheric tumors in two groups characterized by the relative overexpression or downregulation of its gene components. These groups were reminiscent of the G3 (more proliferative) and G1 (less proliferative) groups identified with aGBM signature. Of note, the G3-like group comprised most of the hemispheric tumors with mutant H3.3 histone, whereas wild-type, *BRAF*, and *IDH1* mutated pHGGs were in the G1-like subgroup. Supporting its relevance for the classification of sus-tentorial/thalamic tumors, none of the well-characterized signatures of cell cycle and/or DNA repair described in Materials and Methods provided a better segregation pattern than our 28-gene signature, resulting instead in more heterogeneous grouping (data not shown). When the DIPG cohort was considered, the pHGG signature again identified G1-like and G3-like groups (this latter comprising a little more H3.3 *K27M* mutated tumors), as well as a third group of tumors displaying a less defined gene expression profile, reminiscent of the G2 group identified in aGBM (Figure S4).

### 3.3. DNA Repair Pathways Have a Prognostic Impact in pHGG

**Figure 4:**
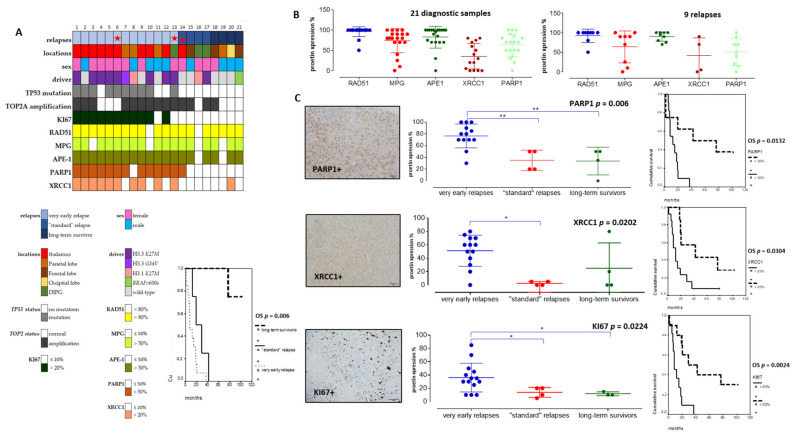
Immunohistochemical analyses in a tissue-microarray collection of 21 pHGGs and their significant correlations with relapses and overall survival (OS). (**A**) Heatmap of clinical, molecular, and protein expressions in the 21 pHGGs associated to a Kaplan Meier analysis of the overall survival discriminating the different subgroups (based on relapsing time). (**B**) Analyses of RAD51, MPG, APE1, XRCC1, and PARP1 protein expressions in 21 pHGG diagnostic samples and 9 paired relapses. (**C**) Comparative analyses of PARP1, XRCC1, and KI67 expressions within each treatment response group, together with an example of their histological immunostaining and Kaplan Meier analyses illustrating their significant impact on overall survival (OS). * and ** signs are underlining statistical significances with a *p* below 0.05.

As our pHGG gene signature confirmed the predominant involvement of several DDR pathways in clustering groups of pHGGs, we set out to validate a selection of its components at the protein level, using the cohort of pHGGs described in Table 1, for which we also determined key molecular drivers. Based on time to relapse, we generated three subgroups of pHGGs comprising, respectively, 13 patients presenting a very early relapse, four patients presenting “standard” relapse times, and four patients considered as long-term survivors (above two years) (Figure 4A). The long-term survivors either bore the *BRAFv600e* mutation (one patient) or displayed LGG-like omic expression (three patients) (data not showed). This clinical subgrouping based on sensitivity to first line therapy was significantly linked to overall survivals (OS curves in Figure 4A, *p* = 0.006). Using TMA slides from this cohort, we analyzed the protein expression levels of components of the HR, BER, NHEJ, and DNA single-strand break (SSB) repair pathways (e.g., RAD51, MPG, XRCC1, APE1, and PARP1). We choose those markers as they are used in routine setting in pathology departments and were linked to the DNA repair pathways exposed in both pediatric and adult signatures. We also examined the proliferation marker KI67 and combined those analyses to *TP53* status, as well as *TOP2A* amplification. The clinical, protein, and molecular results obtained with this pHGG cohort are summarized in Figure 4A and Table 1.

**Figure 5:**
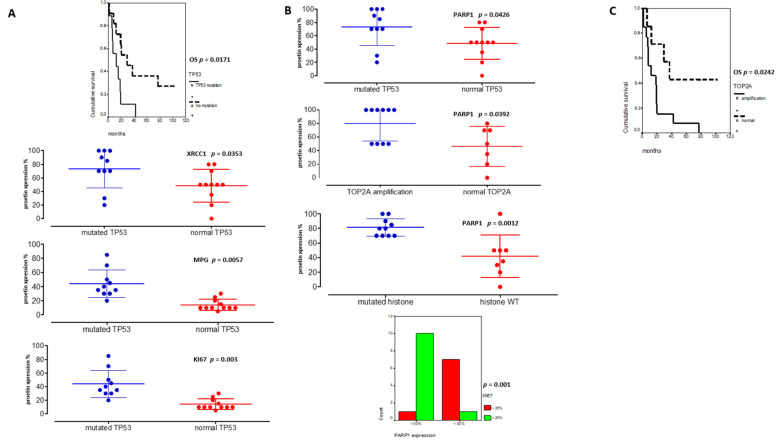
*TP53* and PARP1 are major theranostic markers in pHGGs. (**A**) Significant associations between *TP53* mutated pHGGs and DD repair protein hyper-expressions (%) (XRCC1, MPG and KI67) and their significant involvement on worst overall survival (OS). (**B**) Significant correlations between PARP1 hyper-expression and *TP53* mutation, TOP2 amplification, histone mutations, and KI67 expression (above 20%). (**C**) Kaplan Meier curves reporting the significant impact of TOP2A amplification on pHGG OS.

Among DNA repair proteins, RAD51, MPG, and APE1 were consistently hyper-expressed in all pHGG tumors, both at diagnosis and relapse (Figure 4A,B). Statistically significant differences were observed when the expression levels of these three proteins were compared with that of XRCC1 and PARP1 (Figure 4B, *p* = 0.001 and *p* = 0.006, respectively). Representative immunostainings are presented in Figure S5. Notably, our analysis of XRCC1 and PARP1 suggested the existence of two groups of patients with low or high expression of these proteins. We then did comparative analyses using non-parametric Kruskal–Wallis tests (Figure 4C) to compare XRCC1 and PARP1 expression levels in the three subgroups of our cohort. Notably, PARP1 and XRCC1, as well as KI67, were significantly overexpressed in the subgroup displaying very early relapse and this high expression correlated with a worst OS (Figure 4C). Upregulation of PARP1 in this subgroup was also observed at the mRNA level, as assessed by RT-qPCR (data not showed). Of note, *TP53* mutation was present in 10/21 pHGGs and was significantly associated with a worst outcome and hyper-expression of XRCC1, MPG, KI67, and PARP1 (Figure 5A,B).

On the other hand, PARP1 protein expression was also correlated to *TOP2A* amplification (*p* = 0.0392) and high KI67 expression levels (upper 20%) in Figure 5B (*p* = 0.001). Furthermore, high PARP1 expression level (upper 50%) was the unique biomarker associated to histone mutated tumors (Figure 5B, *p* = 0.0012). Additionally, the presence of a *TOP2A* amplification was linked to a worst outcome (Figure 5C). Finally, in multivariate analyses, *TP53* mutation and PARP1 protein expression remained significant (*p* = 0.0556, HR: 0.64, CI95%: 0.320–0.855; *p* = 0.014, HR: 0.67, CI95%: 0.26–0.671).

### 3.4. PARP1 as a Novel Theranostic and Therapeutic Target in Histone Mutated pHGGs

**Figure 6:**
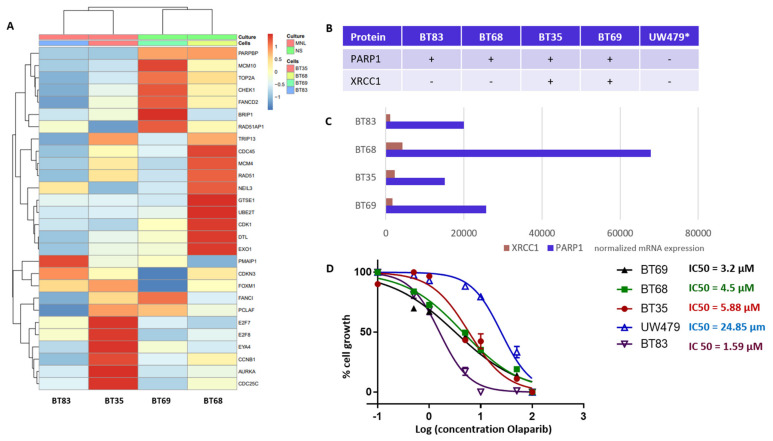
DNA repair and cell cycle gene deregulation studied in patient-derived cell lines (PDCL) and PARP1 functional inhibition. (**A**) Heatmap of standardized gene expression for BT83, BT35, BT69, and BT68 cell lines, based on the pHGG gene signature. (**B**) Basal levels of protein expression detected by immunohistochemical analyses in the PDCL paired tumors and in the UW479 cell line (*, based on previous publication [30]) in absence of Olaparib treatment. (**C**) Normalized mRNA expression levels of XRCC1 and PARP1 in each cell line in the absence of Olaparib, as assessed by RNA sequencing. (**D**) Response of each cell line to Olaparib as determined by a 96 h growth assay, and related IC50 calculations.

Our TMA analyses identified PARP1 and XRCC1 as theranostic markers linked to a worst outcome and a particularly de novo IR-resistant subgroup (displaying a very early relapsing time). As PARP1 overexpression was associated to histone mutated pHGGs, we next characterized four H3.3 *K27M* mutation-bearing PDCLs generated from tumor collection and a more recent diagnosis (BT83). The BT35, BT69, and BT68 PDCLs are indicated by a red star in the upper panel of Figure 4A. They were representative of PARP-expression groups 1 and 2. BT83 patient is part of group 1. A heatmap of the 4PDCL’s RNA sequencing is presented in Figure 6A and is showing standardized profiles of the pHGG signature. BT83 is the cell line, where we have a majority of pHGG signature gene under-expression. Of note, all cell lines showed high *PARP1* protein (Figure 4A or Figure 6B) and RNA expression levels (Figure 6C). In contrast, no correlation between mRNA and protein expression levels was observed for XRCC1 as illustrated by the fact that XRCC1 protein was not present in BT68 (Figure 6B) although it displayed the highest XRCC1 mRNA expression level (Figure 6C). We examined the cellular response of these cell lines to the PARP1 inhibitor Olaparib, using automated microscopic assessment (IncuCyte©) at 96 h of culture. BT83, in which the level of expression of the genes from the pHGG signature was the lowest and where little or no XRCC1 mRNAs were detected, displayed the greatest sensitivity to Olaparib, with a IC50 (half maximal inhibitory concentration) <2 µM. In contrast, the other cell lines showed a higher IC50 ranging from 3.2 to 24.85 µM (Figure 6D), which correlated in part with the co-expression of PARP1/XRCC1 proteins. We also explored the UW479 commercialized cell line where the absence of PARP1 expression previously described explained its resistance to Olaparib treatment [30].

## 4. Discussion

pHGGs are inevitably recurring leading to a fatal issue due to de novo and/or acquired therapeutic resistance. Most pHGGs carry well-identified mutational drivers and up to 60% of all pHGG harbor a somatic heterozygous mutation in histone proteins, a percentage that varies according to the tumor exact location. Given the high chemo- and radioresistance of pHGG tumors, past and recent publications have addressed the links between alterations in DNA repair pathways and pHGG cell behavior [11,12,17,18,19], revealing that DNA damage repair dysregulations were a significant feature of several subgroups of pHGG, including DIPGs. Similar observations were done in adult glioblastomas. In this context, we reasoned that predictive signatures might afford the characterization of pHGG subgroups based on DNA repair and cell cycle gene expression variations [12,17]. We started with previously established signatures of adult glioblastomas, composed of key effectors of the cell cycle as well as components of crucial DNA repair factors [22]. Although our data show that the signatures established in aGBMs can indeed capture features related to cell cycle and DNA repair in pHGG tumors, the observed segregation did not correlate with underlying driver mutations affecting histones, as shown in previous studies [18,20,21,31,32,33]. Our efforts to identify a specific DNA repair and cell cycle gene expression signature of pHGGs led to a 28-gene expression signature that was able to cluster the sus-tentorial and thalamic pHGG group effectively. Of note, we were able to link histone mutated tumors to a more proliferative group displaying upregulation of the gene components of our signature. Previous studies have shown that the H3.3 *G34* mutations impact several aspects of the DDR [20,21,34]. Intriguingly, we could not segregate H3.3- and H3.1-mutated DIPG. Nevertheless, as previously described in Werbrouck et al., 2019, when changes in protein expression levels were investigated, we were able to segregate the population based on DNA repair protein expression and *TP53* mutations. In particular, we noted that PARP1 and XRCC1 protein expression levels as well as alterations in two factors involved in cell cycle modulation were associated to diagnostic and recurrent pHGGs. Several publications [11,35,36,37] have uncovered DDR dysregulations associated to deregulations of DNA repair and chromatin factors like PPM1D or BMI-I. On the other hand, functional studies have revealed that depletion of either component of the H3K36me3: p75 axis could affect the balance between the HR and NHEJ DSB repair pathways [34,38]. This phenotypic diversity could explain in part why H3.3 and H3.1 mutated DIPG could not be segregated as, globally, our cohort contained more histone mutated tumors with potential DDR deregulations comparatively to wild-type, *BRAFv600*, or *IDH1* mutants. In wild-type tumors as well as other driver mutants and certain types of histone mutated pHGG, where the DNA repair genes appear to be comparatively downregulated, it is possible that some of the genes of our signature undergo transient upregulation in response to standard therapy like IR. Finally, other determinants may underlie the segregation seen with our cohorts. Indeed, alterations in DNA repair gene expression have recently been associated to glycolytic metabolism and mTor signaling, which is frequently upregulated in all pHGGs [39].

Our results also demonstrate that PARP1 plays a key role in the very early relapse patients in addition to XRCC1. In addition to being a theranostic and prognostic pHGG marker, PARP1 might also constitute a therapeutic target, e.g., for inhibition by Olaparib. Our results confirmed previous preclinical work in DIPG [33,34], as the preclinical activity of Olaparib in thalamic and DIPG H3.3 mutated tumors demonstrated an efficient response (IC50 < 2 µM) only in the cell line were the genes of our pHGG signature displayed important overall down-regulation. The balance between PARP1 and XRCC1 expression might modulate response to PARP1. Previous studies in other cancer types have reported the efficient targeting of PARP1 in XRCC1 deficient lines [40,41,42]. In our study, the most sensitive cell to PARP1 blockade, e.g., BT83, displayed PARP1 expression and concomitant absence of XRCC1 protein/mRNA expressions. Thus, single targeted therapy trials may only encompass a minority of pHGG and combination therapies should be considered in tumors displaying deregulated DDR.

Finally, a recent study [33] has revealed the association of various DNA repair vulnerabilities in DIPG with the inhibition of intra-tumor immune cells. The DD repair studies in pHGG evidence the transversal role of its deregulation in de novo therapeutic resistance, as well as its impact on pHGG brain microenvironment.

## 5. Conclusions

Collectively, our results highlight the possibility to stratify pHGGs with a specific DNA repair and cell cycle gene signature, and reveal how subsets of pHGGs, especially thalamic and sus-tentorial pHGGs, display specific alterations in the expression of DNA repair and cell cycle genes. Validation studies at the protein expression level provide evidence that PARP1 hyper-expression, associated to XRCC1 expression, *TP53* mutations and *TOP2A* amplification, might represent a new theranostic and therapeutic target in pHGGs.

## Figures and Tables

**Table 1:** Patients’ clinical and theranostic characteristics.

Patients’ Characteristics	Numbers
Age (years)	
mean	12.09
range	8–18
Sex	
female	12
male	9
DIPG	3
thalamic pHGG	10
sus-tentorial pHGG	8
frontal	3
parietal	4
occipital	1
Histone status	
*wild type (wt)*	8
*H3.3 K27M*	9
*H3.1 K27M*	2
*H3.3 G34V*	1
*BRAFv600e*	1
TP53 mutated tumors	10
Classification based on treatment response	
very early relapse (group1)	13
“standard” relapse (group 2)	4
long-term survivors (group 3)	4
EFS (event-free survival) (month)	
median	7
range	1–77
OS (overall survival) (months)	
median	18
range	1–102
Deceased	18
Alive	3

## Data Availability

Gene expression data of adult glioblastomas and its corresponding signature are available in the ArrayExpress database (https://www.ebi.ac.uk/arrayexpress (accessed on 6 April 2021)) under accession number E-MTAB-6425. The 6 pHGG and 10 pilocytic astrocytoma omic data used for the pHGG signature are part of the CIT (Carte d’Identité des Tumeurs) program and will be obtained upon request until 01 January 2022, when they will be published on public omic database. The DIPG and HERBY protocol are already accessible on public database [24,25]. The cell lines data were published on the Gene Expression Omnibus website (www.ncbi.nlm.nih.gov/geo/ (accessed on 6 April 2021)) with the following accession number: GSE101799.

## Supplementary Materials

The following are available online at https://www.mdpi.com/article/10.3390/cancers13092252/s1, Figure S1: Adult glioblastoma (aGBM) signatures, Figure S2: Heatmap of hierarchical clustering in the DIPG cohort based on the 27-gene aGBM signature (A) and ICA (Independent Component Analysis) of cell cycle gene biological processes in the DIPG cohort (B,C), Figure S3: Heatmaps of hierarchical clustering in the sus-tentorial pHGG cohort with the 27-gene aGBM signature, Figure S4: Heatmap illustrating the partial segregation of DIPG specimens from a cohort containing H3.1 *K27M* and H3.3 *K27M* mutant samples based on the specific pHGG 28-DDR signature, Figure S5: Immunohistochemical analyses. Examples of PARP1, XRCC1, KI67, RAD51, MPG, and APE1 positive immunostainings.
