# Supplementary material for: A DNA Repair and Cell Cycle Gene Expression Signature in Pediatric High-Grade Gliomas: Prognostic and Therapeutic Value
Source: Cancers (Basel). 2021 May 7;13(9):2252. doi: 10.3390/cancers13092252 (PMC8125831; doi:10.3390/cancers13092252)

Supplementary Figure 1. Adult glioblastoma (aGBM) signatures. Lists and description of the genes composing the 52 DNA repair and cell-cycle gene expression signature identified in aGBMs as well as its refined 27-gene signature.

A

**AURKA**
AURKB
**CDC25C**
CDC6
**CCNB1**
CCNA2
**CDK1**
CENPA
CENPF
**KIAA0101**
MKI67
MAD2L2
PLK1
**PTTG1**
**TOP2A**
POLK
POLM
POLQ
POLD1
REV1
RFC1
EME1
MUS81
BRCA2
HMMR
MRE11A
PALB2
RAD17
**RAD51**
RAD54B
RAD54L
RAD50
RPA2

DDB2
ERCC3
ERCC4
XPA
XPC
NEIL1
NEIL2
NEIL3
UBE3B
**UBE2T**
UBE3B
**FANCD2**
FANCF
ATR
MSH3
PMS1
RECQL5
SMUG1
NUDT1

B

**AURKA**
AURKB
**CDC25C**
CDC6
**CCNB1**
CCNA2
**CDK1**
CENPA
CENPF
**KIAA0101**
MKI67
PLK1
**PTTG1**
**TOP2A**
POLM
POLQ
EME1
MUS81
**RAD51**
RAD54B
RAD54L
ERCC3
ERCC4
NEIL3
**UBE2T**
UBE3B
**FANCD2**

effectors and regulators of cell cycle
cell cycle and DDR
coordination of DDR
DNA polymerases in NHEJ and DSB
Holliday junction recombination
HR
single-stranded DNA break repair
double-stranded DNA break repair
NER
NHEJ and NER
Fanconi anemia
MMR (MisMatch excision repair)
BER

Supplementary Figure 2. (A). Heatmap of hierarchical clustering in the DIPG cohort based on the 27-gene aGBM signature. Illustrated is the lack of segregation of H3.1 and H3.3 tumors in separate groups based on the DNA damage repair and cell cycle gene expression signature. (**B-C**) ICA (Independent Component Analysis) of the cell cycle component in the DIPG cohort. Weight of the cell-cycle component from ICA of the DIPGs (**B**) showed no linkage with mutations in H3.1 *K27M* and H3.3. *K27M* specimens (**C**).

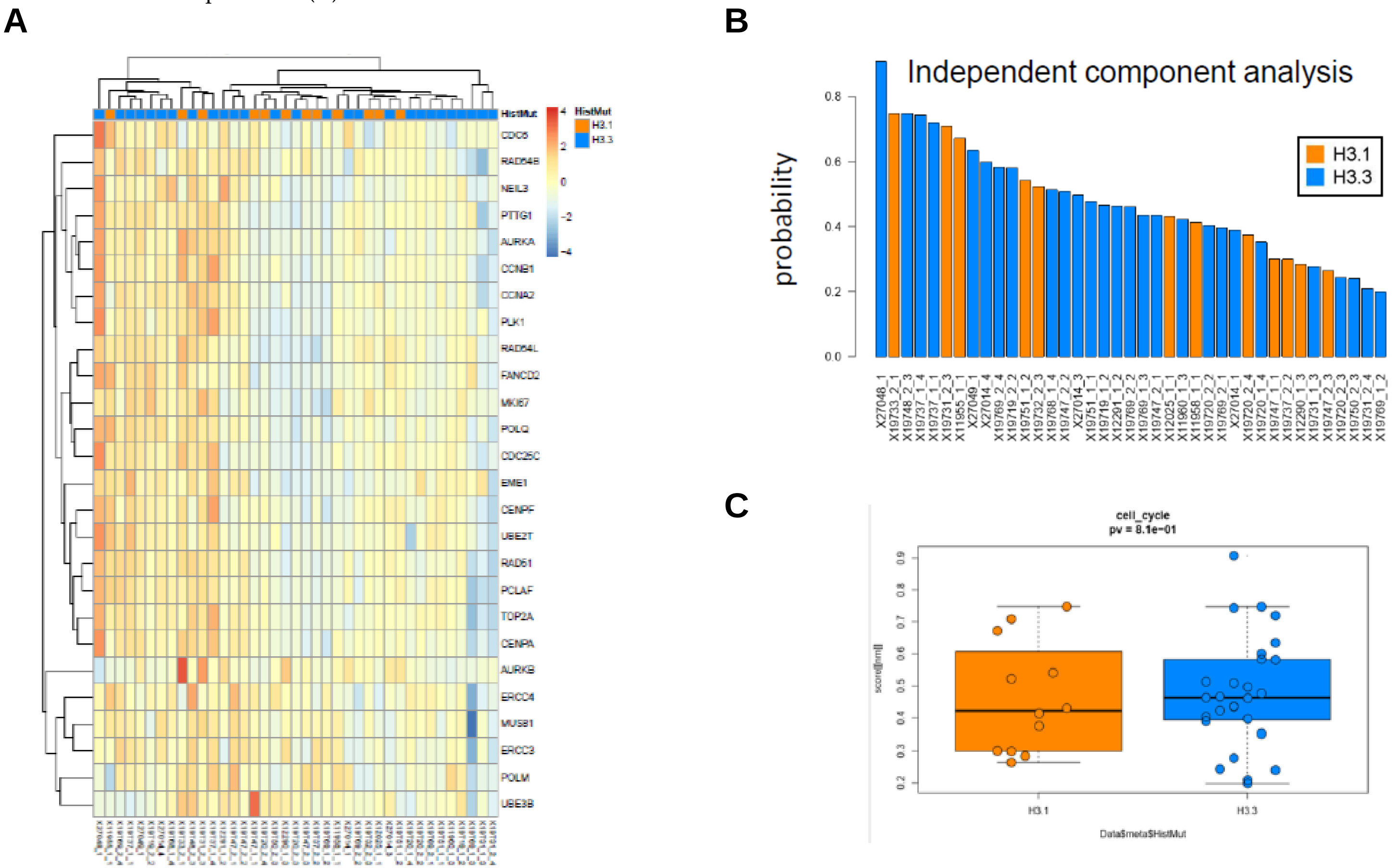

Supplementary Figure 3. Heatmap of hierarchical clustering in the sus-tentorial pHGG cohort based on the aGBM signature. Illustrated is the partial segregation of pHGG specimens into 2 groups based on the 27 DNA damage repair and cell cycle gene expression signature.

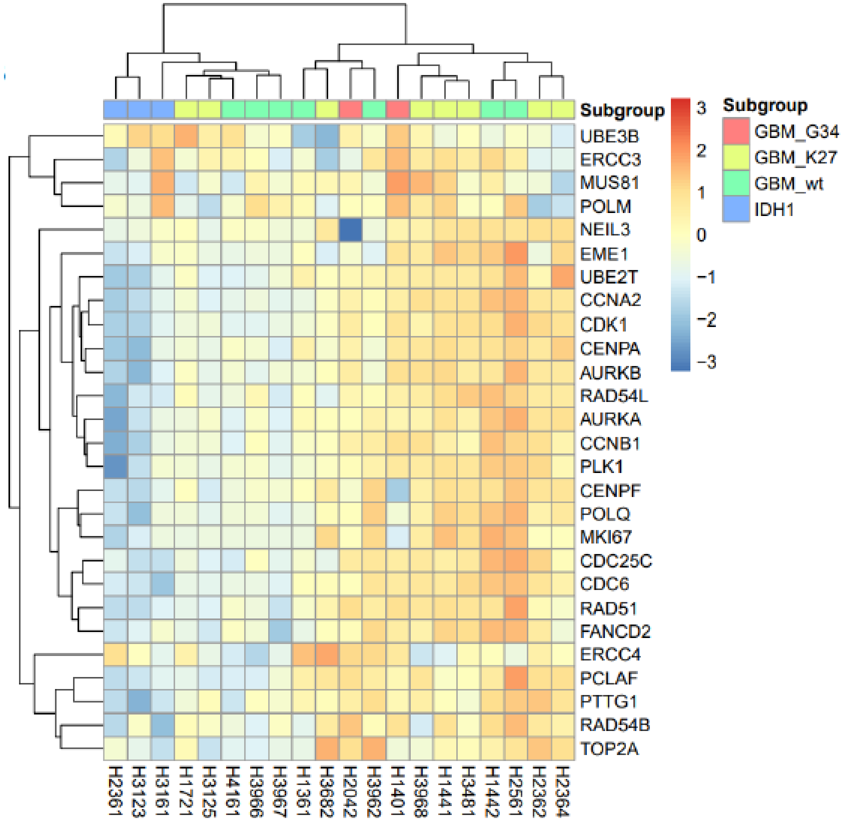

Supplementary Figure 4. Heatmap illustrating the partial segregation of DIPG specimens from a cohort containing H3.1 K27M and H3.3 K27M mutant samples based on the specific pHGG 28-DDR signature.

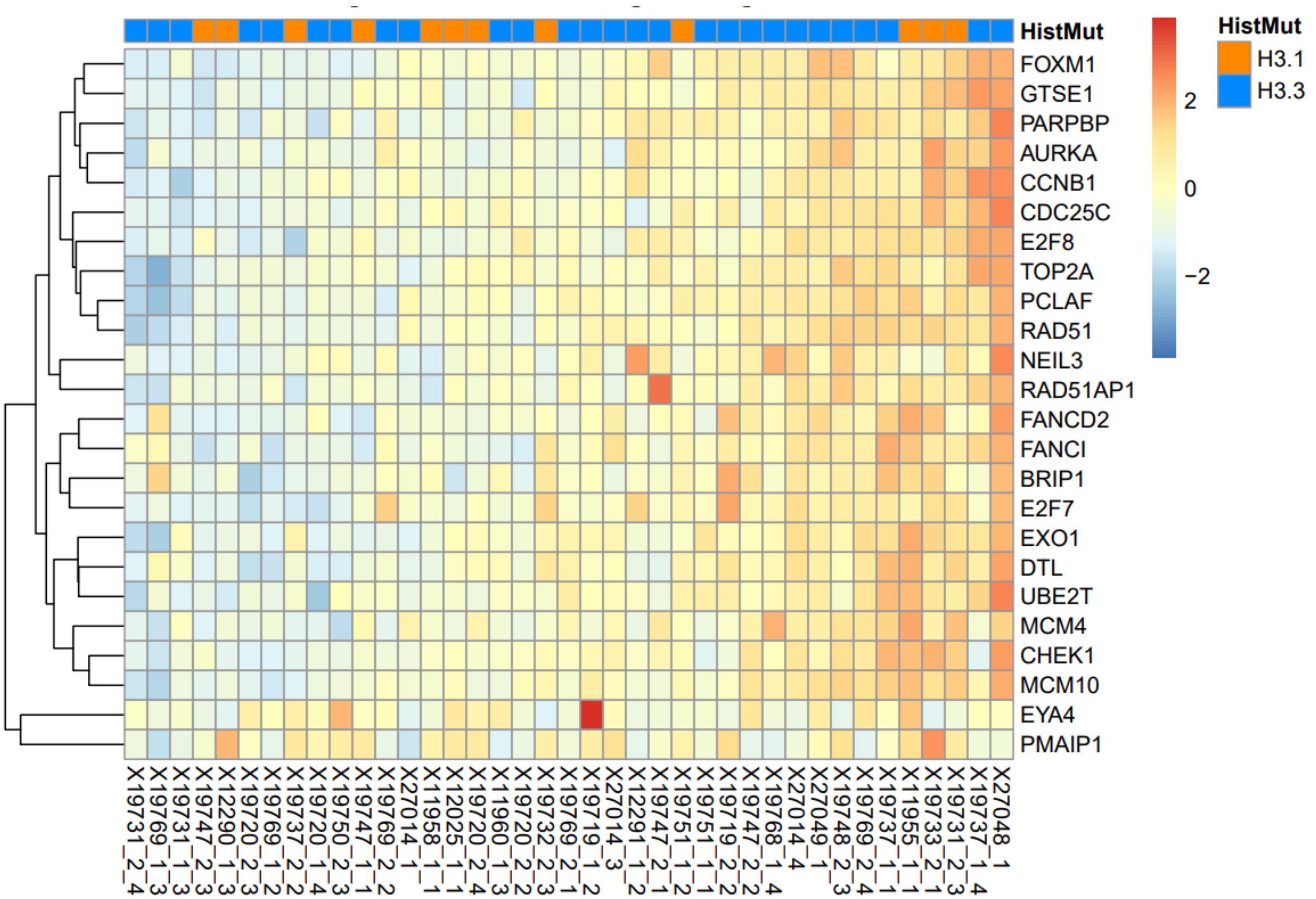

Supplementary Figure 5. Immunohistochemical analyses. Illustration of TMA HE slides and imunostainings on left side. Examples of PARP1, XRCC1, KI67, RAD51, MPG and APE positive immunostainings on right side (x40).

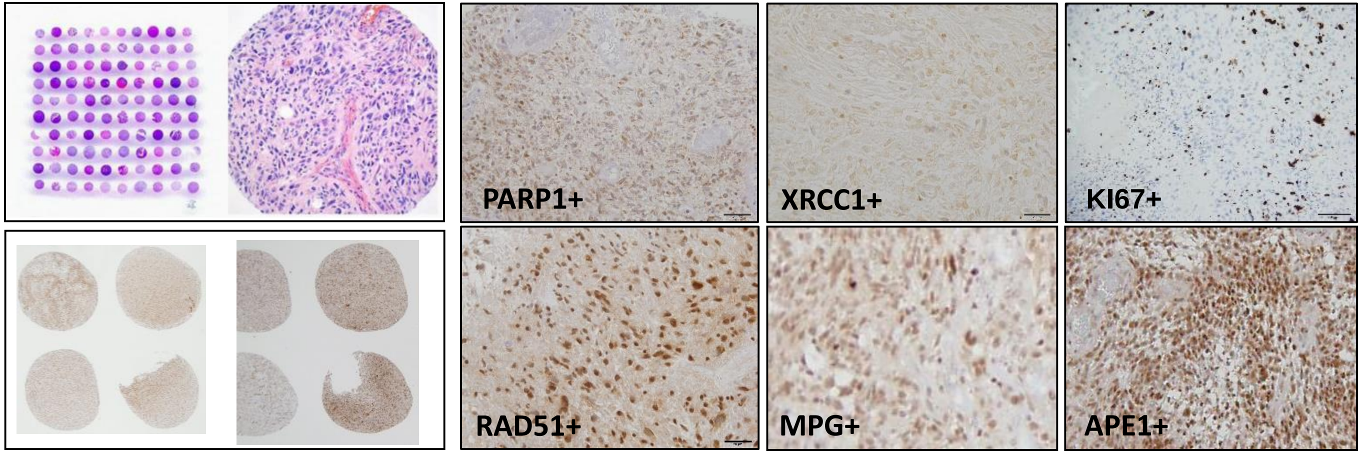

Supplement: Supplementary file 1 [file cancers-13-02252-s001.zip › cancers-1147326-supplementary.pdf]
